# Clinical study of Dendrobium Nobile Lindl intervention on patients with metabolic syndrome

**DOI:** 10.1097/MD.0000000000024574

**Published:** 2021-03-26

**Authors:** Xiaoyan Zhang, Miao Wang, Chengbo Zhang, Zhidong Liu, Shigao Zhou

**Affiliations:** Longhua Hospital, Shanghai University of Traditional Chinese Medicine, Shanghai, China.

**Keywords:** dendrobium nobile, lipid and glucose metabolism, metabolic syndrome

## Abstract

**Background:**

Metabolic syndrome (MS) is a common chronic disease in modern society, and the etiology and pathogenesis of it is still unknown. For its main symptoms: disorder of glucose and lipid metabolism, the usual treatment is applying statin and hypoglycemic drugs. Comparing to the long-term application of these drugs which may cost great side effects, Dendrobium Nobile Lindl (DN) has been proved for its hypoglycemic and lipid-lowering effects without obvious side effects. So this trial is aim to evaluate the efficacy and safety of DN-powder in intervention of MS, and to explore the mechanism of action of DN through multi-group correlation analysis.

**Methods:**

This clinical trial is a single-arm, non-randomized, open, exploratory trial. A total of 30 participants who are suffering from MS will be assigned into therapy group (n = 30). The treatment course will last for 8 weeks, and a follow-up period for 4 weeks. The participants will receive DN-powder for 6 g, twice a day during the study period. The primary outcome will be the change of lipid and glucose metabolism. Other outcomes will be the body weight and body mass index (BMI) which will be assessments record in every 2 weeks. Participants who quit the trial due to untolerable reactions or uncontrollable conditions will enter into a follow-up period after the last treatment. All participants will enter into a follow-up period for 4 weeks after the last treatment. Adverse events will be recorded during the whole study.

**Discussion:**

The results of the trial are aim to provide evidence of the safety and efficacy of DN-powder in intervention of MS which may be potential to become an important alternative therapy for certain patients.

**Trial registration:**

It has been registered at http://www.chictr.org.cn/showprojen.aspx?proj=55914. (Identifier: ChiCTR2000034550), Registered 9 July 2020.

## 1 Introduction

Metabolic syndrome (MS) is a common chronic disease which is characterized by obesity, dyslipidaemia, raised blood pressure and high glucose levels. It can shows hypertriglyceridemia (HTG) and/or low high-density lipoprotein cholesterol in the examination. And it is associated with the risk of type 2 diabetes and developing cardiovascular disease.^[1,2]^

Worldwide, the prevalence of MS usually corresponds to that of obesity and it is quiet different from age, gender, ethnicity and diagnostic criteria. The prevalence of MS was relatively high in Western countries. According to statistics, it was 38% in females and 41% in males in Europe.^[3]^ In the United States, 35% of adults and 50% of people over 60 which 35.6% in females and 30.3% in males were diagnosed as MS.^[4]^ Compared to the prevalence in the Middle East that females ranging from 32.1% to 42.7% while males ranging from 20.7% to 37.2%.^[5]^ In China, the prevalences were 19.2% in males and 27.0% in females while in the population over 60, the number rasied up to 58.1%.^[6,7]^

At present, the etiology and pathogenesis of MS are not completely clear. It is generally believed that central obesity is an initial factor and insulin resistance is the main pathogenesis of MS. Early diagnosis and intervention of MS can reduce the risk of diseases as atherosclerosis, stroke, cerebral hemorrhage even myocardial infarction.^[8,9]^ Regrettably, MS still lacks effective drugs today.

DN in Chinese Medicine is listed as the primary source of medicinal dendrobium in the *Chinese Pharmacopoeia* (2010)^[10]^ and has been used as a dietary additive for hundreds of years. In recent years, scholars have discovered that the chemical components of DN are mainly alkaloids, polysaccharides, flavonoids, phenols, sesquiterpenes, coumarins and steroidal glycoside compounds.^[11,12]^ Polysaccharides and alkaloids are the key to affecting lipid metabolism. Xu et al^[13]^ found that the dried stems of DN contained about 0.41% to 0.64% alkaloids, which was the highest concentration in various dendrobium plants.^[14]^ However the clinical study is still lacking, so we design this exploratory clinical trial to evaluate the safety and efficacy of DN-powder in intervention of MS.

## 2 Methods

### 2.1 Trial design and study setting

The trial is designed as a single-arm, non-randomized, open, exploratory clinical one. The protocol was written in accordance with Standard Protocol Items: Recommendations for Interventional Trials 2013 statement.^[15]^ The study protocol was approved by The Medical Ethics Committee of Longhua Hospital, Shanghai University of Traditional Chinese Medicine (No: 2020LCSY021), and informed consent will be obtained from each participant.

**Figure 1:**
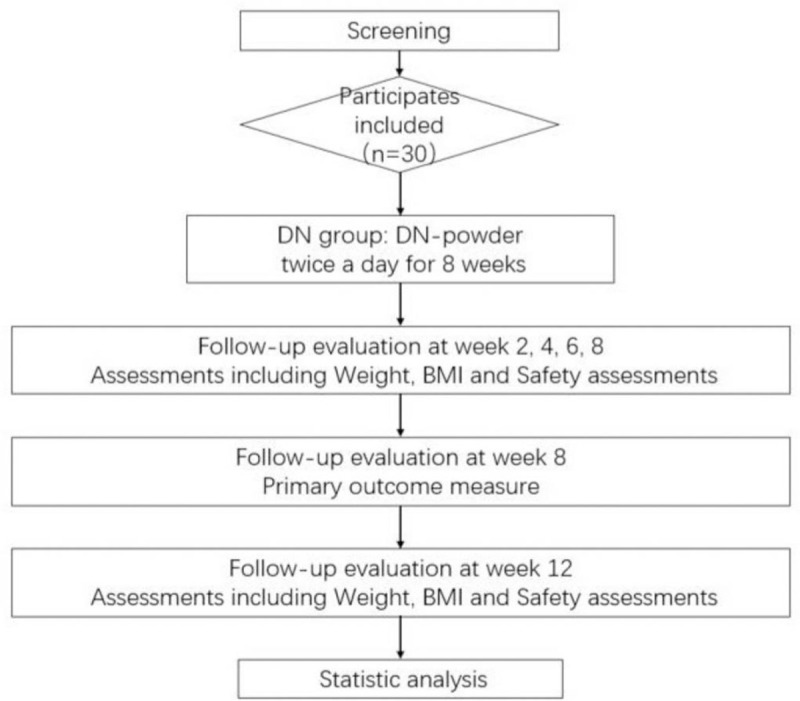
Flowchart.

The trial will be conducted in Longhua Hospital, Shanghai University of Traditional Chinese Medicine, from July 2020 to June 2021 and 30 qualified participants will be recruited from public and will be allocated to the therapy group for 2 months. The flowchart is shown in Figure 1, and the timeline is given in Table 1.

**Table 1:** Timeline.

Study Period						
	Enrollment	Intervention				Close-out
Timepoint	0	Wk 2	Wk 4	Wk 6	Wk 8	Wk 12
Enrollment:						
Informed consent	X					
Inclusion or Exclusion criteria	X					
Baseline information	X					
Allocation	X					
Assessments:						
TG	X				X	
TC	X				X	
HDL-c	X				X	
LDL-c	X				X	
FFA	X				X	
FPG	X				X	
Body Weight	X	X	X	X	X	X
BMI	X	X	X	X	X	X
Safety assessments	X	X	X	X	X	X

BMI = Body mass index, FFA = free fatty acid, FPG = fasting plasma glucose, HDL-c = high-density lipoprotein cholesterol, LDL-c = low density lipoprotein cholesterol, TC = total cholesterol, Wk = week.

### 2.2 Eligibility criteria

#### 2.2.1 Inclusion criteria

Participants who meet all the following criteria will be recruited:

1. Aged between 18 to 65 years old, both genders;
2. Meet the diagnostic criteria of MS:^[16–18]^
  ① Overweight or Central obesity: BMI cut-off point for obesity (>24.0 kg/m^2^) or waist circumference ≥90 cm for males and ≥85 cm for females.
  ② Hyperglycemia: fasting plasma glucose (FPG) ≥6.1 mmol/L (110 mg/dl) or 2 hour plasma glucose ≥7.8 mmol/L (140 mg/dl), or previously diagnosed type 2 diabetes.
  ③ Hypertensive: systolic blood pressure ≥130 or diastolic blood pressure ≥ 85 mm Hg, or treatment of previously diagnosed hypertension.
  ④ Raised TG level: fasting TG level≥1.7 mmol/L (150 mg/dl), or received treatment for this lipid abnormality.
  ⑤ Reduced HDL-c: fasting blood HDL-c<0.9 mmol/L (35 mg/dl) for males and <1.0 mmol/L (39 mg/dl) for females.
  ^∗^with 3 or all of the above 5 items can be diagnosed as metabolic syndrome.
3. Agree to join this clinical study, volunteer to sign up informed consent form and agree to participate in all visits and inspections in accordance with the requirements of the study protocol.

#### 2.2.2 Exclusion criteria

Participants who meet any of the following conditions will be excluded from the study:

1. Pregnant and lactating women;
2. Combined with severe primary diseases, including but not limited to heart, liver, and kidney dysfunction, tumors and other serious cerebrovascular diseases.

### 2.3 Intervention

#### 2.3.1 Selection of DN

The origin of DN is from Guizhou, China. It is processed by Guizhou Natil Biotechnology Co., Ltd. as an agent. The company has the Certificate of Good Manufacturing Practices for Pharmaceutical Products, Drug Production License and Business License form People's Republic of China which will process DN-powder in accordance with the drug guidelines issued by the *Chinese Pharmacopoeia (2010)*.^[10]^ It is made to ensure that the DN-powder the company collects, processes and manufactures can be completely decomposed after being taken by the human body without leaving other chemical substance. Besides, Zunyi Medical University will conduct sampling composition determination, doing quality inspection and supervision of the decoction pieces to ensure that the DN-powder meets the specifications.

#### 2.3.2 Instructions of DN

According to the standard oral administration method of DN published by the *Chinese Pharmacopoeia (2010)*.^[10]^ DN-powder was given a fixed dose of 12 g per day (twice a day, 6 g each time) to the subjects for oral on an empty stomach. 4 weeks is a course of treatment and the trail will take 2 courses in total. During the period, body weight and BMI will be record in every 2 weeks. After the treatment cycle, laboratory tests will be performed. Participants who quit the trial due to untolerable reactions or uncontrollable conditions will enter into a follow-up period after the last treatment. All participants will enter into a follow-up period for 4 weeks after the last treatment. Adverse events will be recorded during the whole study.

#### 2.3.3 Intervention of diet and exercise

##### 2.3.3.1 Diet intervention

Helping participants to establish a healthy diet refer to *Dietary Guidelines For Americans 2015–2020*^[19]^ which including focusing on diversity, nutritional density and quantity of foods; limiting the energy supply of sugars and saturated fatty acids; reducing sodium intake; switching to choose healthy foods and beverages. The total calories intake per day should be 2000 for female and 2400 for male.

##### 2.3.3.2 Exercise prescription

The exercise prescription follows FITT principle which stands for frequency, intensity, time, and type of exercise. According to American College of Sports Medicine guidelines for exercise testing and prescription^[20]^ and requiring participants to do 150 minutes of moderate-intensity aerobic exercise (50%–70% of maximum heart rate; maximum heart rate = 220 - age) every week and record it.

#### 2.3.4 Falling off criteria

For those who have to stop taking the drug due to serious adverse drug reactions; those whose treatment is ineffective and the participants give up to continue the trial; those who have poor compliance, or unwilling to obey the rules, or cannot complete the follow-up; and those who have no adverse reactions during the study, but for other reasons to interrupt the trial (such as immigration, lost follow-up, etc.).

#### 2.3.5 Termination criteria

Participants withdrew their informed consent and asked to quit the trial; pregnancy incidents occurred during the study; side effects could not be tolerated; other situations that the researcher considered participants must quit the trial.

#### 2.3.6 Combination medication statement

After joining the group, participants must maintain a standardized diet and a relatively stable amount of exercise. During the study period, no therapeutic drugs other than those specified in the study should be used; those with other diseases that must taking other drugs should be recorded in detail by the researcher.

### 2.4 Outcome measurements

Outcome will be performed by laboratory tests or physical examination after the intervention (at week 8).

Primary outcome will be the change of blood lipid and glucose which are the most intuitive assessments of the body. Main assessments of blood lipid includes TG, 3total cholesterol, HDL-c, low density lipoprotein cholesterol, free fatty acid and FPG.

Other outcomes are body weight and BMI. Weighing the body weight to see if there is any weight loss. Using the following formula to calculate BMI: Body weight in kilograms divided by height in meters squared. Recording body weight and calculating BMI at each visit, namely every 2 weeks. The evaluation will be performed in baseline and treatment endpoint. In addition, the researchers will keep in touch with participants via phone call, message or through the Internet to ensure the progress every day.

Baseline observation include demographic data (age, gender, occupation, education level), medical history, allergies history, past or combined medication, height, waist circumference. Assessments include clinical laboratory examination (TG, total cholesterol high-density lipoprotein cholesterol, low density lipoprotein cholesterol, free fatty acid and FPG), body weight, BMI, and safety assessments. Adverse events will be recorded throughout the study. Researchers will evaluate the severity of the adverse event and decide whether to continue the treatment or not. Participants with adverse events will receive necessary medical interventions. Participants who quit the study due to untolerable reactions or uncontrollable conditions will enter into the follow-up period after the last treatment. All participants will enter into the follow-up period for 4 weeks at the end of the treatment.

### 2.5 Determination of sample size

The trial is a single-arm, non-randomized, open, exploratory clinical trial. For exploratory and safe considerations, it is designed as a small sample trial. The sample size cannot be calculated due to lack of database. For the determination of participants recruited in this trial, after consulting with experts in this field, we finally designed the sample size to 30 (n = 30).

### 2.6 Statistical analysis

In the trail, Statistical Product and Service Solutions software will be applied to conduct statistical analysis. A full analysis set will be considered as the principal analysis and the per-protocol is set as the subset of full analysis set. The per-protocol will consist of participants who perform the study and have principal variable base-line values in this trial. The principal parameters will be conducted at baseline, each treatment and in the follow-up period.

Descriptive statistics will be used to balance baseline and parametric characteristics. Researchers will compare and analyze the data from baseline to the end of the follow-up period to confirm the difference between primary and other outcomes. When it comes to normal distribution, one-way analysis of variance will be used to compare subjective and semi-objective outcomes. Otherwise, it will be run by rank sum test. In the efficacy comparison stage, the outcome measurements before and after the intervention will adopt single factor analysis. For each outcome, *P* < .05 is considered statistically significant.

Multi-group correlation analysis include extraction and sample derivation of metabolites (blood samples and stool samples). GC-MS analysis Metabolomics instrumental analysis will be performed on an Agilent 7890A gas chromatography system coupled to an Agilent 5975C inert MSD system (Agilent Technologies Inc., CA). Principal component analysis is used to truly reflect the differences between groups, if the differences between groups are small, then the supervised model, namely Partial least squares-discriminant analysis will be used to eliminate interference. It will also adopt Student *t* test to identify differential metabolites and analyze correlation matrix.

### 2.7 Quality control

Main researchers in this trial will be asked for Good Clinical Practice certificates and the other researchers will receive relevant training on the qualifications required for clinical trials to ensure the quality of the research. All researchers will perform operations in accordance with Standard Operating Procedures to make sure the consistency of the research. The test results will be saved in the original documents, laboratory results and records. The original data will be registered in the electronic case report form (e-CRF) and the data uploaded in the e-CRF is parallel to the original data. Kernel researchers will verify the accuracy of the original data. The data will be analyzed independently by different statisticians. The data may be used to do interim analyses, and the monitor will supervise the whole study period and make the final decision if terminate the trial.

All participants will be collected blood samples and stool samples at the research hospital site when participating in the study for the first time. The laboratory will assist in the collection of samples, and the main researchers will collect and evaluate the samples. Longhua Hospital, Shanghai University of Traditional Chinese Medicine will provide sample processing, aliquoting and storage facilities. All samples will be placed in the storage warehouse of the hospital after centrifugal extraction, and stored in a liquid nitrogen refrigerator at -80°C. All samples will be coded with a unique number. Any process involving sample transfer will strictly abide by the sample transfer operating specifications. These processes will be supervised by the Medical Ethics Committee of Longhua Hospital.

### 2.8 Informed consent and data confidentiality agreement

The investigator is responsible for explaining the purpose, methods, benefits and potential risks of this trial to each participant and obtaining informed consent signed by the participants. For participants who are unable to sign the informed consent form by themselves for any reason, it must be signed by their legal representative instead. In addition, in the informed consent form, we attached a clause: consent to allow clinical investigators, research supervisors, ethics committees, and drug regulatory authorities to check the original clinical research data, in order to verify the reliability of the data results. This clause will also obtain the informed consent of the participants.

The investigator must follow the principle of confidentiality when recording the trial data of the participants. When uploading participants’ information into the e-CRF form, 2 researchers will input data independently, and conceal personal information such as the participant's name and address, and protect the participant's personal information before, during, and after the trial. Participants’ medical records (research medical records, physical and laboratory examination reports, etc.) will be kept in the hospital intact, and doctors will record the results of laboratory examination in the participants’ outpatient medical records. Any public report on the results of this trial will not disclose the personal identities of participants. We will make every effort to protect the privacy of participants’ personal information within the scope permitted by law.

Sample information will be electronically transmitted to the Statistics Center for verification and it will be kept strictly confidential. The information provided to researchers will not contain personal identification of participants. Researchers have no access to personal identifiers, nor will they be able to link these test results with personal identifier information. No individual results will be shown in publications or other reports. There are related regulations in the informed consent form. When the participants sign the consent form, they agree to collect and submit additional blood samples and stool samples for storage and future testing. Participants will check it out as an additional item.

### 2.9 Trial status

The trial started in May, 2020 and is currently recruiting. The planned end of the study is at the end of May, 2021.

## 3 Discussion

This study protocol is for a single-arm, non-randomized, open, exploratory clinical trial which aims to evaluate the efficacy and safety of DN-powder in intervention of MS and to explore the mechanism of action through multi-group correlation analysis.

For now, early diagnosis and lifestyle changes are still the first choice to intervene MS. Lifestyle interventions include control, proper exercise, changes in eating habits, smoking cessation and moderate drinking. For those who have taken preventive measures but failed to reduce risk of disease, corresponding medications should be taken for each component.^[21]^ Traditional Chinese Medicine (TCM) has few side effects, high clinical safety, low price and easy access which expands the boundaries of modern medicine.

Recent years, a large number of experimental studies have shown that DN plays an important role in glucose and lipid metabolism. Some studies have shown that DN can reduce the serum levels of alanine aminotransferase and aspartate aminotransferase, make attenuation of malondialdehyde production, and improve ultrastructural morphology in hepatocytes to reduce the liver damage relevant to CCl4. DN is also beneficial to the gene expression of glucose and lipid metabolism which can enhance the expression of genes related to the Nrf2-antioxidant pathway.^[22,23]^ In addition, DN possess anti-oxidative effects, and its total alkaloids can prevent LPS-induced neuroinflammation.^[24]^

This clinical trial will provide information on the efficacy and safety of taking DN- powder orally in the intervention of MS. Not only in terms of physical measurements, but also about biomedical variables. The trial will be conducted on the basis of evidence-based medicine and provide conclusive evidence.

The trial takes a rigorous design that meets clinical requirements. As far as we know, this is the first exploratory trial of taking DN- powder orally in the intervention of MS. The result will be used to verify and carry out other clinical studies on the intervention of TCM in MS.

## Acknowledgments

We sincerely thank SG Z and M W as the corresponding author. We also would like to thank all the researchers who participated in this trial.

## Author contributions

XY Z designed the trial protocol, and analyzed the data together with CB Z. ZD L assisted in translation of original documents.

All authors read and ratified the final draft of the manuscript.

**Conceptualization:** Xiaoyan Zhang.

**Data curation:** Xiaoyan Zhang, Chengbo Zhang.

**Formal analysis:** Chengbo Zhang.

**Resources:** Zhidong Liu.

**Supervision:** Shigao Zhou, Miao Wang.

**Writing – original draft:** Xiaoyan Zhang.
